# Anti-proliferative of physcion 8-O-β-glucopyranoside isolated from *Rumex japonicus* Houtt. on A549 cell lines via inducing apoptosis and cell cycle arrest

**DOI:** 10.1186/1472-6882-14-377

**Published:** 2014-10-06

**Authors:** Qi-Chao Xie, Yu-Peng Yang

**Affiliations:** Department of Oncology, the Second Affiliated Hospital, Third Military Medical University, 183 Xinqiao main street, Chongqing, 400037 China

**Keywords:** Physcion 8-O-β-glucopyranoside, *Rumex japonicus* Houtt, Anti-proliferative activity, Apoptosis, Lung cancer

## Abstract

**Background:**

Lung cancers are leading causes of cancer death, and *Rumex japonicus* has been traditionally used in folk medicine as anti-microorganic, anti-inflammatory and anti-tumor agents. This study was designed to investigate the anti-proliferative activity of physcion 8-O-β-glucopyranoside (PG) isolated from *Rumex japonicus* Houtt. on A549 cell lines.

**Methods:**

In our present study, PG was isolated and identified from the ethanol extracts of *R. japonicus*. MTT method was used to evaluate the anti-proliferative activity of PG on A549 cell lines, and cell cycle distribution assay, apoptosis assay, and western blot analysis *in vitro* were used to explore the possible mechanisms.

**Results:**

From the results of our present study, cell viability was obviously inhibited by PG, in a dose- and time-dependent manner. Our results also suggested that the anti-proliferative effect of PG was related to cell cycle arrest at the G2/M phase through repression of cdc2 and Cyclin B1 protein expression. In addition, the results of apoptosis assay and western blot analysis indicated that the anti-proliferative activity could be related to apoptosis via up-regulating the expressions of Bax, caspase-3 and caspase-7, and down-regulating the expressions of Bcl-2.

**Conclusions:**

In conclusion, the PG has significant anti-proliferative activity on A549 cell lines, and the possible mechanism was related to cell cycle arrest at the G2/M phase, and apoptosis via the regulations of Bax, Bcl-2, and caspase-3 and caspase-7.

## Background

Lung cancers are the leading causes of cancer death worldwide. Lung cancers are commonly classified as small cell lung cancer (SCLC) and non-small cell lung cancer (NSCLC), and NSCLC constitutes approximately 80% of the lung cancer cases [1–3]. It’s well known that smoking is the predominant etiologic risk factor for lung cancers, and polycyclic aromatic hydrocarbons are reported as the major causative agents among cigarette smoke components for lung cancer development [2, 4]. Currently, chemotherapy is still the major therapeutic method for lung cancers after surgery resection, and this treatment can significantly improve the survival rate of patients [5, 6]. However, the long-term survival rate remains low and the 5-years survival rate after surgery is less than 70% [5, 7]. Besides, the synthetic drugs, which have severe side effects, are commonly the only option for cancer chemotherapy [8, 9]. Therefore, it is important to search for new reliable therapeutic agents with low toxicity to lung cancer treatment. Traditional Chinese medicine (TCM) has been used for cancer treatment for a long time either being used alone or in combination with western medicines [10]. And natural products isolated from the plants are important sources of cancer therapies [11, 12].

*Rumex japonicus* Houtt, a perennial herb plant belonging to the family Polygonaceae, is widely distributed throughout China (known as *Yang-Ti*, in Chinese). It has been traditionally used in folk medicine as anti-microorganic, purgative, anti-inflammatory and anti-tumor agents [13–15], and *R. japonicus* contains a large number of anthraquinones, oxanthrones, and flavones [13, 14, 16]. However, there has been no report regarding the active anti-tumor compounds isolated from *R. japonicus* and its possible mechanisms thus far. Physcion 8-*O*-*β*-glucopyranoside (PG), one of the major bioactive compounds presented in the *R. japonicus*, is an anthraquinones. The aim of the present study is to investigate the anti-proliferative activity of PG based on our preliminary experiments.

## Methods

### Plant material

*Rumex japonicus* Houtt was purchased from the Chuqimen Market of Traditional Chinese Herbs and identified as the roots of *R. japonicus* by the department of Traditional Chinese Medicine of the Second Affiliated Hospital, Third Military Medical University. A vouncher specimen of *R. japonicus* (No. RJ2010-09) was deposited at the Second Affiliated Hospital, Third Military Medical University.

### Cells culture

Human lung cancer cell line A549, human Myeloid Leukemia cell Line IL-60, human breast cancer cell line MCF-7, colonic carcinoma cell Line HCT-8, human cervical carcinoma cell line Hela, and human hepatoma cell line HepG2 were purchased from American Type Culture Collection. The cells were cultured in RPMI-1640 medium (Gibco, CA, USA) with 10% fetal bovine serum (Gibco, CA, USA) and antibiotics (100 U/mL penicillin and 100 μg/mL streptomycin). The cell line was kept at 37°C in 5% CO_2_/95% air.

### Chemicals

Silica-gel (100–200, 200–300 mesh) was purchased from Qingdao Haiyang Chemical Co., Ltd. (Qingdao, China); methanol (MeOH) [analytical reagent grade (AR)], Petroleum ether (AR), ethyl acetate (AR), n-butanol (AR) were purchased from Sinopharm chemical reagent Co., Ltd. (Shanghai, China); Sephadex LH-20 was purchased from *H&E* Co., Ltd. (Beijing, China). The RPMI 1640 media and fetal bovine serum (FBS) were purchased from Invitrogen (Carlsbad, USA). Methyl-thiazdyldiphenyl-tetrazolium bromide (MTT) and dimethyl sulfoxide were purchased from Sigma (NY, USA). Human phospho-Cdc2 (Tyr15), Cdc2, Cyclin B1, caspase-3, caspase-7, Bax and Bcl-2 monoclonal antibody and Annexin V/FITC kit was purchased from Beyotime Institue of Biotechnology (Haimen, China). All other chemicals used in this study were of analytical reagent grade.

### Extraction, isolation and preparation of total extracts and pure compounds

Dried *R. japonicus* (50 kg) was powdered and extracted with 75% aqueous ethanol by reflux three times (each extraction period lasted 3 h). The solvent was evaporated under vacuum to afford crude total extract of *R. japonicus*. Then the extract was suspended in water and partitioned with petroleum ether, chloroform, ethyl acetate, and aqua-saturated *n*-butanol successively. The *n*-butanol fraction was subjected to repeated column chromatography over silica gel (100–200 mesh) column chromatography and eluted with ethyl acetate-methanol (20:1 ~ 1:2). Then, similar fractions were combined based on the basis of thin layer chromatography analysis (TLC), and 7 fractions (I-VII) were obtained. By using a series of chromatographic techniques, such as silica gel column chromatography (200–300 mesh) and Sephadex LH-20 chromatography, the compound was isolated from fractions III (140 mg) [16, 17].

### MTT reduction assay

Cells (1 × 10^4^/0.2 mL) were seeded in 96-well plates and treated on the following day with PG ranged from 5 μg/ml to 80 μg/ml (for 24 or 48 h, respectively). After that, MTT assay was carried out [9] and optical density (OD) was read at 570 nm using a 96-well plate reader. Since reduction of MTT only occurred in metabolically active cells, the level of activity was a measure of the viability of the cells. The inhibition rate was calculated according to the following formula: (OD_control_ – OD_treatment_)/OD_control_ × 100%. The IC_50_ (inhibitor concentration at which occurs 50% inhibition of proliferation) value was calculated using Bliss method by SPSS software.

### Cell cycle distribution assay

A549 Cells (5 × 10^5^/mL) were seeded in 6-well plates. On the following day, the cells were treated with 20, 40 and 80 μg/mL of PG for 24 h, or treated with 80 μg/mL of PG for 12, 24, 48 h, respectively. Then, cells were trypsinized, washed with Phosphate Buffered Saline (PBS) and fixed in 1 mL of ice-cold 70% ethanol overnight at 4°C. The cells were concentrated by removing ethanol and treated with 0.01% Dnase-free RNase A for 10 min at 37°C. Cellular DNA was stained with 0.05% propidium iodide (PI) for 20 min at 4°C in darkness. The cell cycle distribution was detected with flow cytometry (FCM) on a FACScalibur cytometer (Becton Dickinson, US). The percentage of cells at G_1_, S, or G_2_/M phase was documented using ModFit software (Becton Dickinson, US).

### Apoptosis assay

Two methods were used for apoptosis assays. Firstly, A549 Cells (5 × 10^5^/mL) were seeded in 6-well plates. On the following day, the cells were treated with 20, 40 and 80 μg/mL of PG. 48 hours later, cells were trypsinized, washed with PBS and stained using an Annexin V/FITC kit. The cells were detected with flow cytometry (FCM) on a FACScalibur cytometer (Becton Dickinson, US).

Secondly, the cells (1 × 10^4^/0.2 mL) were seeded in 96-well plates and then treated with 100 nM of Ac-DEVD-CHO (caspase inhibitor) [18] for 3 h. The cells were treated with PG (20, 40 and 80 μg/mL) for 48 h. Cells survival was determined by MTT assay to evaluate whether the anti-proliferative effect of PG was related to apoptosis through caspase pathway that lead to apoptotic.

### Western blot analysis

A549 Cells (5 × 10^5^/mL) were seeded in 6-well plates. On the following day, the cells were treated with 20, 40 and 80 μg/mL of PG. 48 hours later, cells were harvested and homogenized with lysis buffer. Total cell protein was extracted and the protein concentration was determined using BCA Protein Assay Kit (Beyotime Institue of Biotechnology, Haimen, China). Subsequently, the total cellular protein was separated by 12% SDS-polyacrylamide gel electrophoresis (SDS PAGE), and transferred to PVDF membranes (Millipore USA). Membranes were blocked with 5% fat-free milk in TBST (10 mM Tris–HCl, 0.1 M NaCl_2_, 0.1% Tween 20, pH 7.4), and incubated with phospho-Cdc2 (Tyr15), Cdc2, Cyclin B1, caspase-3, caspase-7, Bax and Bcl-2 monoclonal antibodies, respectively. Protein bands were detected by incubating with HRP-conjugated secondary antibodies (Beyotime Institue of Biotechnology, Haimen, China) and visualized by BeyoECL plus Kit, and then immunoblotting signals were scanned and quantitated using a ChemiDoc XRS gel imaging system (Bio-Rad USA). Fold changes of protein levels were determined by using Bio-Rad quantity one software and compared to the protein expression of β-actin.

### Statistical analysis

All results were presented as mean ± standard deviation (SD) from at least three independent experiments. Statistical analyses were performed using the two-tailed Student’s *t*-test. Differences with the p value less than 0.05 was considered statistically significant.

## Results

### IC_50_ values of the total extract of R. japonicus on the six human cancer cell lines

The investigation on anti-proliferative activities of the total extract *from R. japonicus* was conducted, and the results are represented in Table 1. The data demonstrated that, the total extract displayed moderate anti-proliferative activities against IL-60 and HepG2 cell lines (IC_50_ were 127.43 ± 3.12, and 146.25 ± 3.97 μg/mL, respectively); furthermore, no obvious anti-tumor effect was observed on the MCF-7, HCT-8, and Hela cells (IC_50_ > 150 μg/mL); however, the total extract showed a significant cytotoxic activity on the A549 cell line with the IC_50_ less than 50 μg/mL (IC_50_ = 44.19 ± 1.08 μg/mL).

**Table 1:** **IC**_**50**_ **of the total extracts of** ***Rumex japonicus*** **Houtt**

Cells	IL-60	A549	MCF-7	HCT-8	Hela	HepG2
**IC**_**50**_ **(μg/mL)**	127.43 ± 3.12	44.19 ± 1.08	>150	>150	>150	146.25 ± 3.97

Cells were treated with PG for 48 h, and the MTT assay were determined to obtain the IC_50_.

### Analysis of the compound isolated from R. japonicas

**Figure 1:**
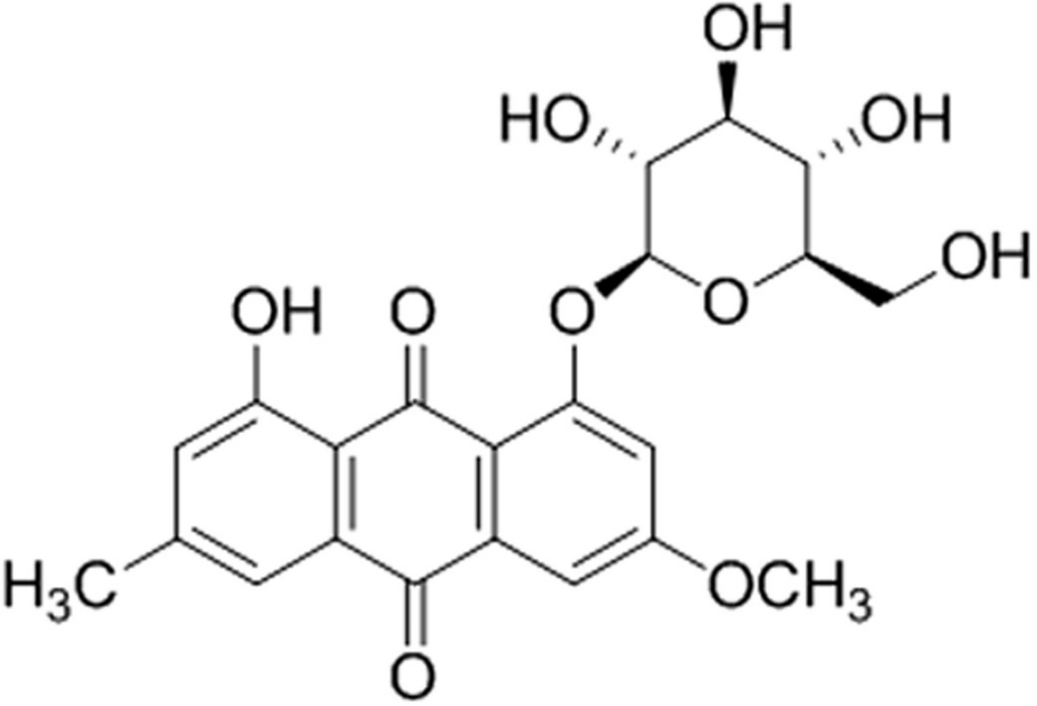
**Molecular structure of physcion 8-** ***O*** **-** ***β*** **-glucopyranoside.**

The compound isolated from the *Rumex japonicus* Houtt. was identified as physcion 8-*O*-*β*-glucopyranoside [16, 17] (Figure 1, Table 2). The spectral data of the compound were as follows: Yellow needle crystals; melting point (m.p.) 231-233°C; electrospray ionisation tandem mass spectrometry (ESI-MS) *m/z*: 445.1 [M - H]^-^, and it showed the molecular ion at *m/z* 446, which was in accordance with the molecular formula C_22_H_22_O_10_. The ^1^H-NMR and ^13^C-NMR information was shown in Table 2.

**Table 2:** ^**1**^ **H NMR (600 MHz) and** ^**13**^ **C NMR (150 MHz) data of compound 1 in DMSO-** ***d***_***6***_ **(δ, ppm)**

Position	δ _H_ (J in Hz)	δ _c_	Position	δ _H_ (J in Hz)	δ _c_
1	12.87 (s, -OH)	161.6	8a		114.5
2	7.17 (brs)	125.1	9a		114.4
3		147.1	10a		136.4
4	7.46 (d, 1.6 Hz)	119.3	1′		100.9
5	7.38 (d, 2.6 Hz)	106.7	2′		73.3
6		164.7	3′		77.2
7	7.19 (d, 2.6 Hz)	107.5	4′		69.8
8	3.74 (s)	161.1	5′		76.8
9		186.4	6′		60.8
10		181.7	3-CH_3_	2.39 (s)	21.3
4a		132.1	6-OCH_3_	3.97 (s)	56.1

### Physcion 8-O-β-glucopyranoside inhibits the viability of A549 cells in dose- and time-dependent manners

**Figure 2:**
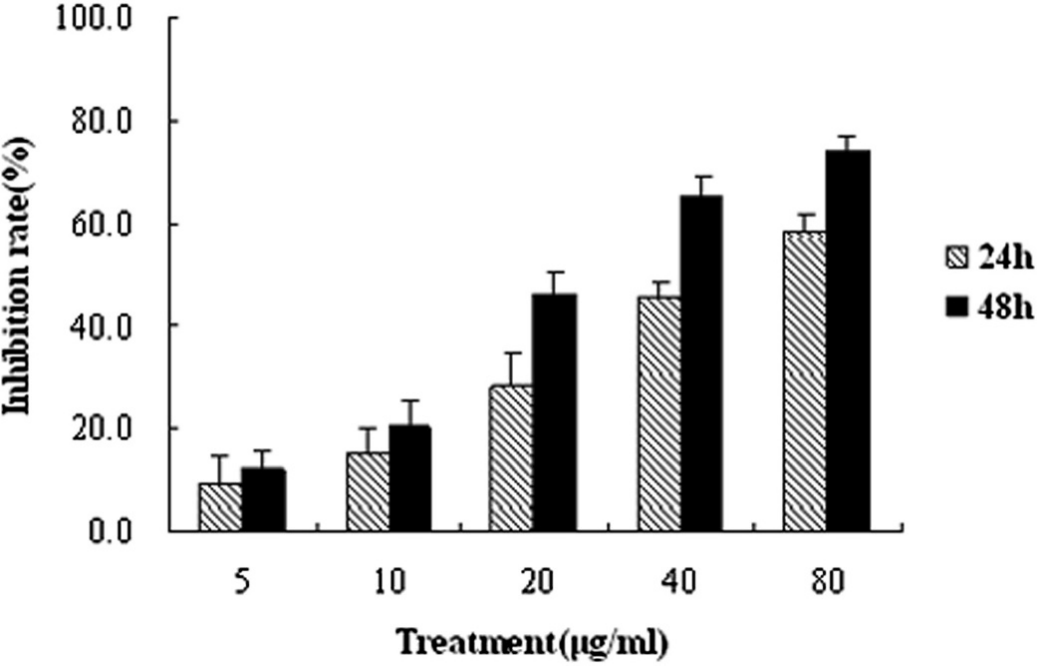
**PG inhibits the viability of A549 cells in dose- and time-dependent manners.** In the figure, Physcion 8-O-β-glucopyranoside abbreviated as PG. Cells were seeded in 96-well plates and treated on the following day with indicated concentrations of PG for 24 and 48 h, respectively, the cells viabilities were determined by MTT assay.

To evaluate the cytotoxic effect of PG on A549 cell and get the IC_50_ value, the viability of cells treated with different concentrations (ranging from 5 to 80 μg/mL) of PG was measured using MTT test. The result showed that PG reduced cell survival in a dose- and time-dependent manner (Figure 2). The IC_50_ of PG to A549 cell lines was 53.01 μg/mL at 24 h and 27.31 μg/mL at 48 h, respectively. Based on the results, the concentration of 20, 40 and 80 μg/mL were selected and used in the subsequent experiment.

### PG induced cell cycle arrest at G_2_/M phase in dose- and time-dependent manners

**Figure 3:**
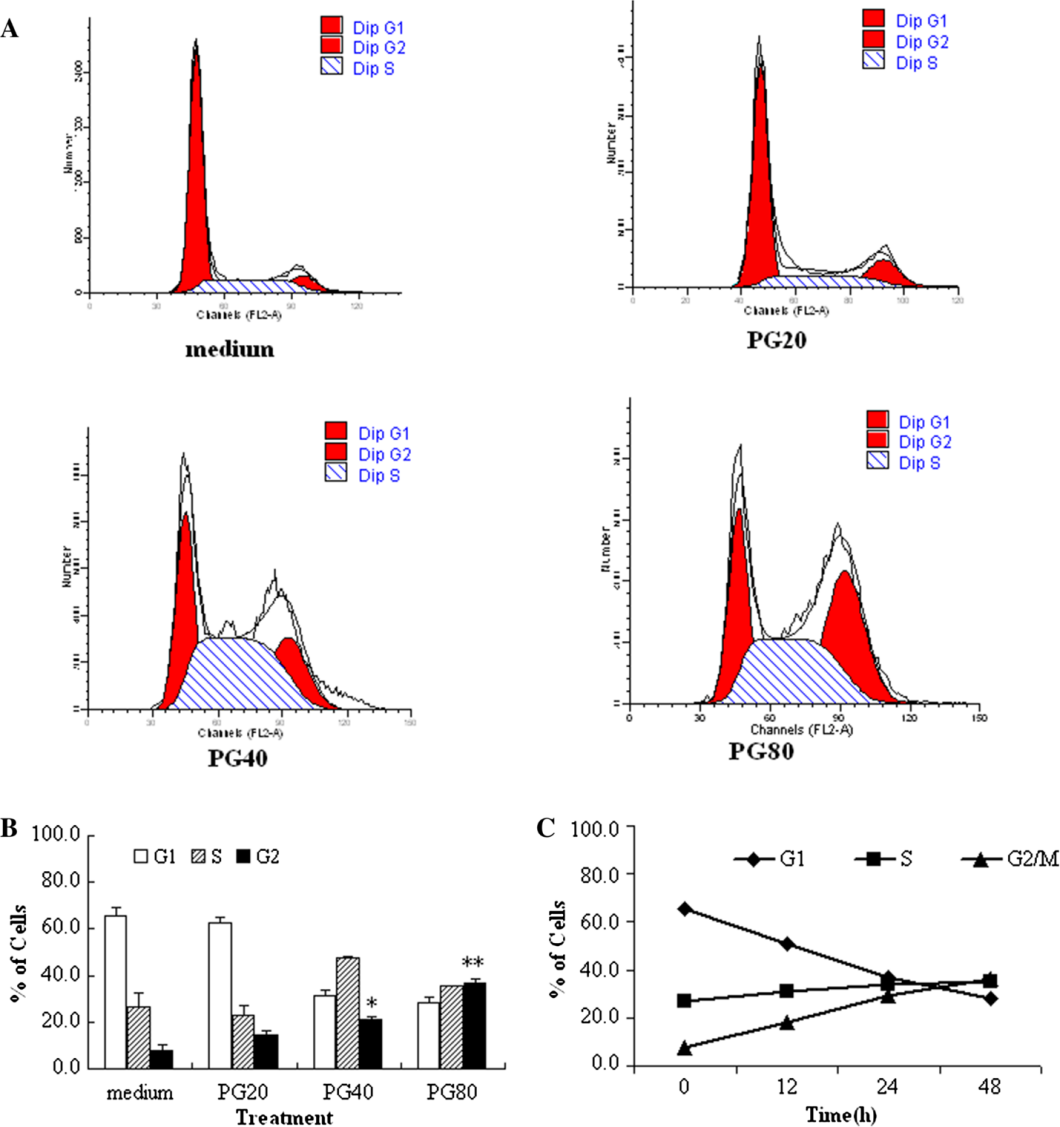
**Cell cycle distribution of A549 cell** ***in vitro*** **.** In the figure, 20, 40 and 80 μg/mL of PG abbreviated as PG20, PG40 and PG80, respectively. **(A and B)** PG induced cell cycle arrest at G_2_/M phase in dose-dependent manners. Cells were treated with 20, 40 and 80 μg/mL of PG for 24 h, respectively, and then cells were harvested to determine cell cycle distribution by flow cytometry. *p < 0.05 *vs.* medium; **p < 0.01 *vs.* medium. **(C)** PG induced cell cycle arrest at G_2_/M phase in time-dependent manners. Cells were treated with 80 μg/mL of PG for 12, 24, 48 h, respectively, and then cells were harvested to determine cell cycle distribution by flow cytometry.

Cell cycle arrest was the major reason of cell death induced by anti-tumor drugs. Cell cycle regulation was important for cell proliferation [19–21]. To examine whether the anti-proliferative effect of PG was related to cell cycle arrest, the cell cycle distribution was tested. The results showed 20, 40 and 80 μg/mL of PG had significantly affected cell cycle distribution, leading to cell cycle arrest at G_2_/M phase. Cell amount at G_2_/M phase increased from 7.7% (medium group) to 14.6% (20 μg/mL, PG), 20.8% (40 μg/mL PG), and even up to 36.9% (80 μg/mL PG). Cells in the G_1_ or S phase shifted concomitantly to the changes detected in G_2_/M (Figure 3). The results also showed that treatment of cells with 80 μg/mL PG resulted in cell cycle arrest at G_2_/M phase in a time-dependent manner*.* Cell amount at G_2_/M phase increased from 8.4% (12 h) to 19.3% (24 h), even up to 36.5% (48 h). The results suggested the antitumor effect of PG was related to cell cycle arrest at the G_2_/M phase.

### Effect of PG on the expression of G_2_/M cell cycle regulatory proteins

**Figure 4:**
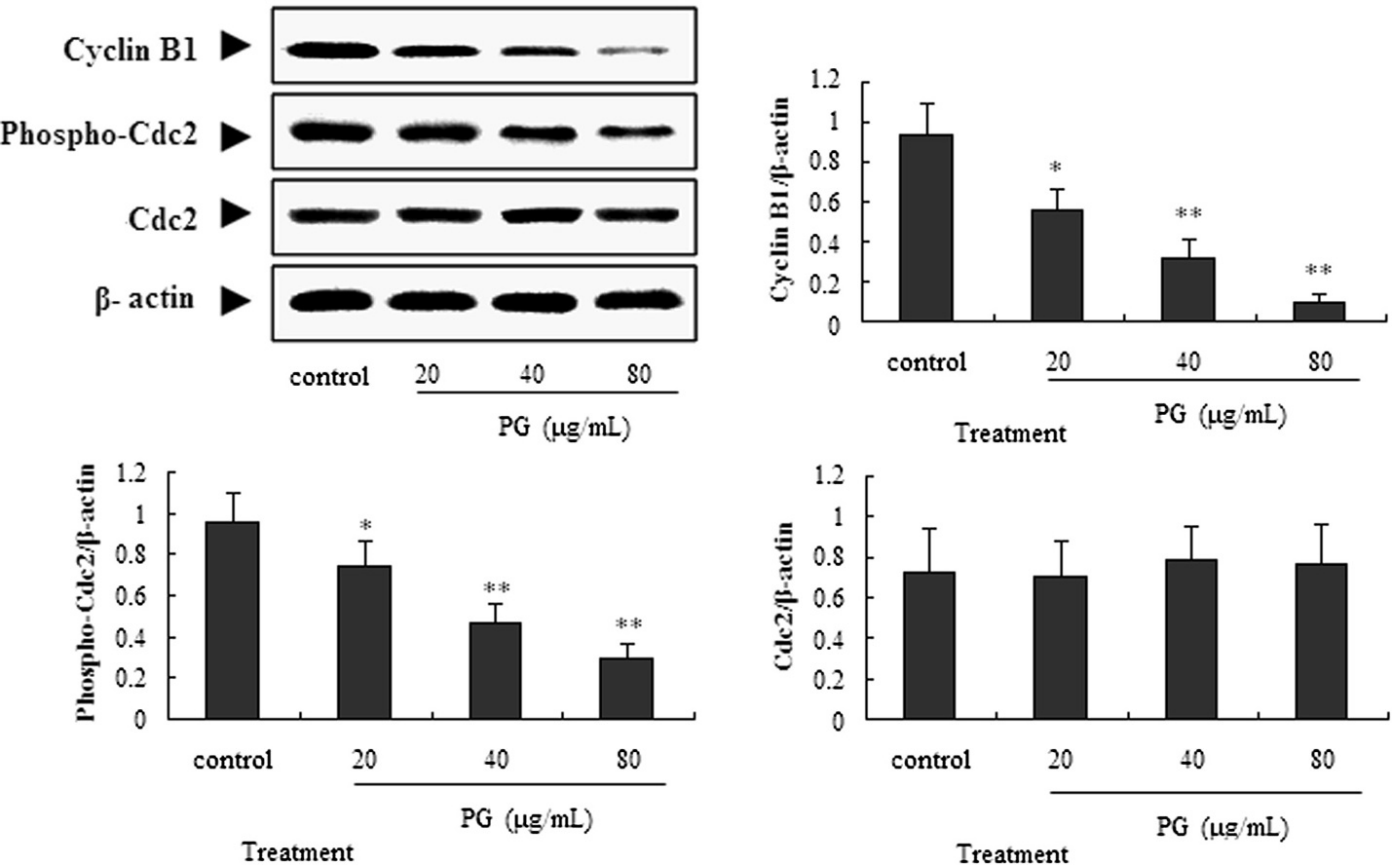
**Effect of PG on the cyclin proteins of Cdc2 and cyclin B1.** In the figure, 20, 40 and 80 μg/mL of PG abbreviated as PG20, PG40 and PG80, respectively. *Physcion 8-O-β-glucopyranoside* abbreviated as PG. A549 cells were treated with PG (20, 40 and 80 μg/mL) for 48 h, and then the cells were collected for western blot assay *in vitro*.

Our data showed that PG-mediated anti-proliferative effects were accompanied by cell cycle arrest at G2/M phase. Cell cycle progression through G2/M is regulated principally by the sequential activation of the Cyclin B/Cdc2. To explore the mechanism by which PG induced the cell cycle arrest at the G2/M phase in A549 cells, we used western blot assay to determine if PG modulate the expression of G2/M cell cycle regulatory molecules. The results demonstrated that treatment of A549 cells with 20, 40, 80 μg/mL of PG for 48 h resulted in a 0.6, 0.34, 0.08 fold decrease in expression of Cyclin B1 and a 0.78, 0.49, 0.33 fold decrease of phospho-Cdc2 (Tyr15) compared to control group (Figure 4). On the other hand, total levels of Cdc2 remained largely unchanged after treatment (Figure 4). These data suggested decreased cyclin B1 and cdc2 protein expressions contributed to G2/M arrest induced by PG in A549 cells.

### PG induced A549 cell apoptosis

**Figure 5:**
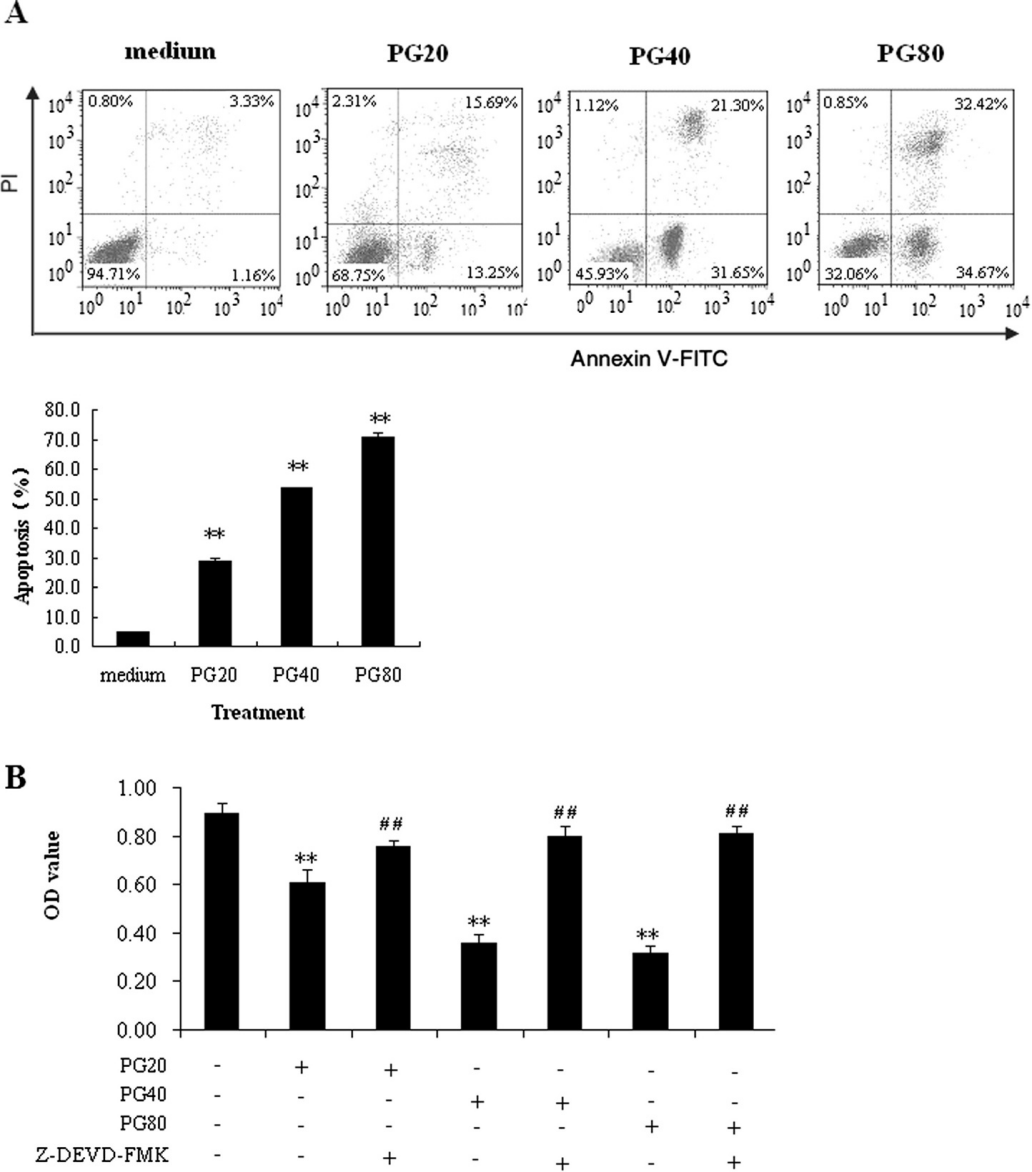
**Apoptosis of A549 cell in vitro.** In the figures, Ac-DEVD-CHO abbreviated as Ac, 20, 40 and 80 μg/mL of PG abbreviated as PG20, PG40 and PG80, respectively. **(A)** The cell Apoptosis was determined by flow cytometry. The cells were treated with 20, 40 and 80 μg/mL of PG. 48 hours, and then cells were trypsinized, washed with PBS and stained using an Annexin V/FITC kit. The cell apoptosis was detected with flow cytometry. Fluorescence intensity for annexin V/FITC is plotted on the x-axis, and PI is plotted on the y-axis. **(B)** The cells were treated with or without Ac-DEVD-CHO for 3 h, then incubation with 20, 40 and 80 μg/mL of PG for 48 h, then MTT assay was carried out. **p < 0.01 *vs.* medium; ^# #^ *p* < 0.01 *vs.* PG.

In order to make sure whether the anti-proliferative effect of PG on A549 cell was due to apoptosis, the flow cytometry analysis was used. Our results revealed PG could significantly increase apoptosis of the cells compared to medium. 20, 40 and 80 μg/mL of PG produced a 26.9%, 53.7% and 70.72% of propidium iodide (+)/annexin V (+) cells (necrosis/late apoptosis), respectively, However, the medium group only produced 4.86% of cells (necrosis/late apoptosis) (Figure 5A). To further confirm 20, 40 and 80 μg/mL of PG indeed increased apoptosis, Ac-DEVD-CHO, a caspase inhibitor [22], was used. The results further demonstrated PG in combination with Ac-DEVD-CHO could partly reverse the anti-proliferative effect compared to PG-alone treatment group (Figure 5B).

### *PG induce apoptosis*via *Caspase pathway*

It is well known that most cell apoptosis-inducing factors eventually cause cell apoptosis through the caspase-mediated signal transduction pathway. Caspase 3 and caspase 7 is the executioner caspases in the caspase cascade that lead to apoptotic [22].

**Figure 6:**
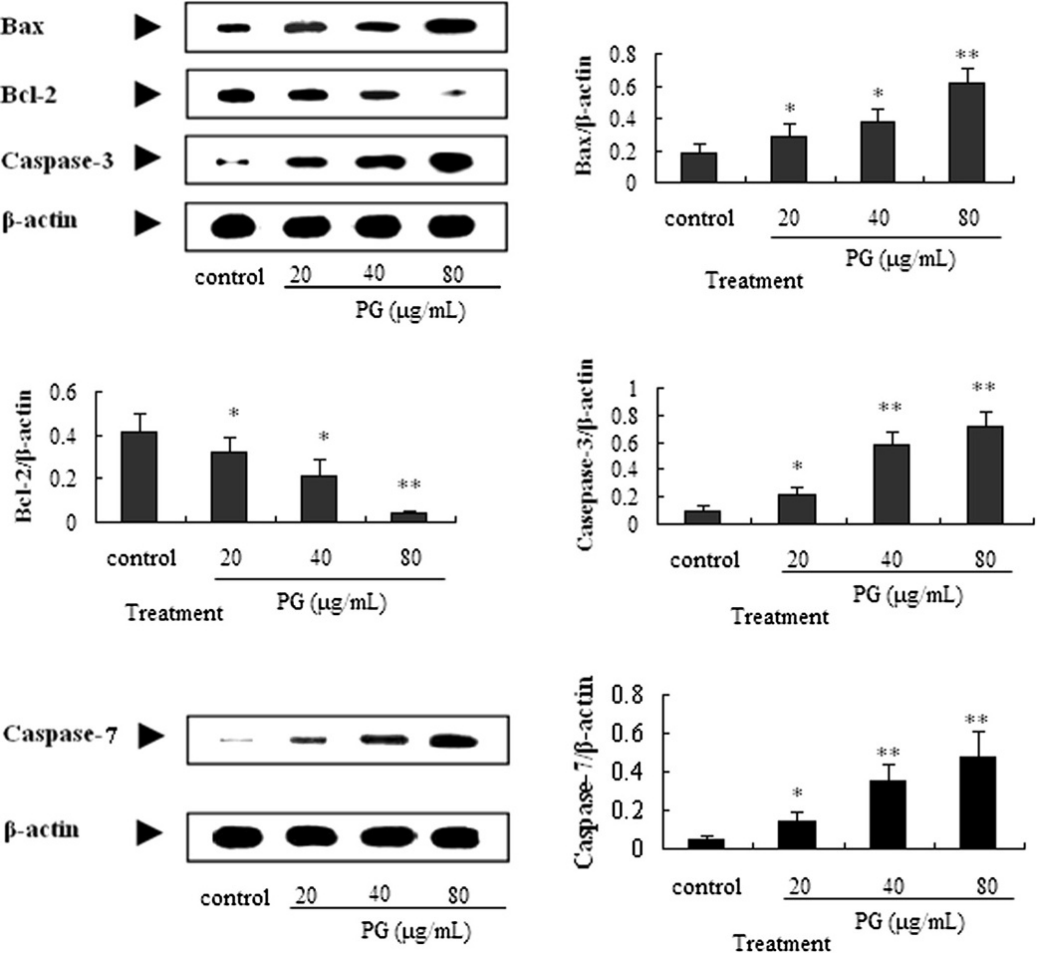
**The protein expression of caspase-3, Bax, Bcl-2 and caspase-7 by western blot assay** ***in vitro.*** In the figure, 20, 40 and 80 μg/mL of PG abbreviated as PG20, PG40 and PG80, respectively. A549 cells were treated with 20, 40 and 80 μg/mL of PG for 48 h, then the cells were collected for western blot assay *in vitro*. *p < 0.05 *vs.* control; **p < 0.01 *vs.* control.

Bax is a well-known pro-apoptotic protein and Bcl-2 is an anti-apoptotic protein, the ratio of Bcl-2/Bax has a decisive role to induce apoptosis [23, 24]. To determine whether the apoptosis was regulated by caspase-3, caspase-7, Bax and Bcl-2 proteins, we measured the expression of caspase-3, caspase-7, Bax and Bcl-2 proteins in A549 cells treated with 20, 40 and 80 μg/mL of PG using Western blot analysis. Our data showed that after 20, 40 and 80 μg/mL of PG treatment, the cleaved Caspase-3 were increased 2.1, 5.8, 7.2 fold, the cleaved caspase-7 were increased 2.8, 7.0, 9.6 fold, and the protein expression of Bcl-2 level was decreased, but the Bax expression level was increased, when compared to the medium group (Figure 6). Therefore, the ratio of Bcl-2/Bax was significantly increased after PG treatment, when compared with the medium. All results showed that cell apoptosis is also a major reason of PG anti-proliferative effect, and the Caspase-3, Caspase-7, Bcl-2 and Bax are involved.

## Discussion

Nowadays, cancer is recognized as a chronic and refractory disease that markedly affects patient quality of life. Furthermore, cancer is an important cause of substantial medical care expenditures. Currently, approximately 40% cancer patients with stage III or IV NSCLC currently can not be resected, and chemotherapy is the major treatment in such cancer patients [5]. However, the prolonged use of these drugs can induce the unbearable side-effects. Therefore, how to find the novel treatment strategies against NSCLC cancer with less adverse side-effects became a serious and urgent problem.

As a part of our continuing study regarding investigating the anti-cancer agents derived from TCMs, this study was designed to examine the effect of PG on cell proliferation, cell cycle, and apoptosis in A549 cells. To the best of our knowledge, this is the first report regarding anti-tumor activity of PG. Interestingly, the present study showed that PG significantly inhibited cell proliferation in dose-dependent manners and this inhibitory effect resulted from cell cycle arrest at G2/M phase. Additionally, we also found that exposure of A549 cells to PG resulted in an obvious increase in apoptosis, suggesting that apoptosis and cell cycle arrest contribute to growth inhibition in PG-treated A549 cells. G2 to M phase progression is regulated by a number of proteins of the CDK/cyclin family; importantly, the activation of the Cdc2/Cyclin B1 complex is required for transition from G2 to M phase of the cell cycle [25]. To elucidate the mechanism by which PG-induced cell cycle arrest at G2/M phase, we determined the expressions of phospho-Cdc2 (Tyr15) and Cyclin B1 in A549 cells treated with PG. The results of our present study showed that PG markedly down-regulated the expressions of Cyclin B1 and the phosphorylation of Cdc2 at Tyr15, suggesting that repression of cdc2 and Cyclin B1 is likely to be involved in PG-induced G2/M arrest. Collectively, the results of our study indicated that PG treatment could potentially inhibit the proliferation of A549 cells by cell cycle arrest at G2/M phase.

Apoptosis is one of the major mechanisms of cell death in response to cancer therapies, and caspases proteins play the key roles in the execution phase of apoptosis. Caspase 3 and caspase 7 are the executioner caspases in the caspase cascade that lead to apoptosis. Caspase-3 is a caspase protein that interacts with caspase-8 and caspase-9, encoded by the CASP3 gene Caspase-3 has been activated in the apoptotic cell both by extrinsic (death ligand) and intrinsic (mitochondrial) pathways. The activated Caspase-3 induces Caspase-8 activation, then actives Bax. Bax activates Cyt c, and induces apoptosis. Our results showed that Caspase-3, caspase-7 and Bax expressions were up-regulated by treatment with PG, whereas the protein expression level of Bcl-2 was down-regulated. These findings above demonstrate the mechanism by which PG exert their growth inhibitory effect, is related to induce A549 cells apoptosis through inducing caspases pathway and cell cycle arrest at G2/M phase.

## Conclusions

In conclusion, A549 cells viability was significantly inhibited by PG *in vitro*, in a dose- and time-dependent manner, and the possible mechanism may be related to cell cycle arrest at the G_2_/M phase, and apoptosis might be via the regulations of Bax, Bcl-2, Caspase-3 and Caspase-7. Consequently, the present results indicated that the PG is worthy for further exploration for lung cancer treatment, and more laboratory investigations are necessary to elucidate the mechanism of actions.
